# TACE Plus Lenvatinib Versus TACE Plus Sorafenib for Unresectable Hepatocellular Carcinoma With Portal Vein Tumor Thrombus: A Prospective Cohort Study

**DOI:** 10.3389/fonc.2021.821599

**Published:** 2021-12-23

**Authors:** Biao Yang, Luo Jie, Ting Yang, Mingyang Chen, Yuemei Gao, Tian Zhang, Yuzu Zhang, Hao Wu, Zhengyin Liao

**Affiliations:** ^1^ Department of Gastroenterology, West China Medical School, Sichuan University, Chengdu, China; ^2^ Department of West China School of Public Health, Sichuan University, Chengdu, China; ^3^ Department of Abdominal Oncology, West China Medical School, Sichuan University, Chengdu, China; ^4^ Department of Stomatology, Hospital of Stomatology, Sichuan University, Chengdu, China; ^5^ West China Medical School, Sichuan University, Chengdu, China

**Keywords:** hepatocellular carcinoma, lenvatinib, sorafenib, portal vein thrombosis, transcatheter arterial chemoembolization

## Abstract

**Background and Objectives:**

This study aimed to compare the efficacy of transarterial chemoembolization (TACE) plus sorafenib (TACE-S) to TACE plus lenvatinib (TACE-L) for the treatment of HCC with portal vein tumor thrombus (PVTT).

**Methods:**

This cohort study recruited patients from September 2017 to September 2020. A total of 59 and 57 consecutive patients were treated with TACE-L and TACE-S, respectively.

**Results:**

Before propensity score matching (PSM), comparing TACE-L to TACE-S, the median overall survival (OS) time was 16.4 months and 12.7 months, respectively [hazard ratio (HR) 1.34; 95% confidence interval (CI): 0.81–2.20; *p* = 0.25]. The median progression-free survival (PFS) time was 8.4 months and 7.43 months, respectively (HR 1.54; 95% CI: 0.98–2.41; *p* = 0.081). After PSM, the median OS time was 18.97 months and 10.77 months, respectively (HR 2.21; 95% CI: 1.12–4.38; *p* = 0.022); the median PFS time was 10.6 months (95% CI: 6.6–18.0 months) and 5.4 months (95% CI: 4.2–8.1 months), respectively (HR 2.62; 95% CI: 1.43–4.80; *p* = 0.002). After PSM, the overall response rate (ORR) was 66.8% vs. 33.3% [odds ratio (OR) 0.85; 1.05–6.90; *p* = 0.037].

**Conclusion:**

Both TACE-L and TACE-S are safe, well-tolerated treatments for HCC with PVTT. In HCC with PVTT, TACE-L was significantly superior to TACE-S with respect to OS, PFS, and ORR. A larger-scale randomized clinical trial is needed.

## Introduction

Hepatocellular carcinoma (HCC) is the fifth most frequent cancer worldwide (1). Unfortunately, 30%–62% of patients with HCC tend to develop portal vein tumor thrombus (PVTT), which is a poor risk factor for overall survival (OS) (2).

For patients with HCC and PVTT, sorafenib is recommended as the first-line treatment by the Barcelona Clinic Liver Cancer (BCLC) staging system and European Association for the Study of the Liver (3, 4). Unfortunately, HCC with PVTT treated with sorafenib only achieved a 5.6 months of OS time (2). Peng et al. (5) demonstrated that combining TACE with sorafenib (TACE-S) significantly prolonged median OS compared to mono-sorafenib (16.0 vs. 10.0 months). Similarly, the corresponding time to progression (TTP) was longer in the TACE-S group than the mono-sorafenib group (10.0 vs. 5.0 months, *p* < 0.001). According to the PVTT classification validated by Yamada et al. (6) in patients with right or left PVTT (Vp3), the median OS in the TACE-S group and mono-sorafenib group was 11.0 months and 7.0 months, respectively (*p* = 0.001). The corresponding TTP was 6.0 months and 3.5 months in the TACE-S group and mono-sorafenib group, respectively (*p* < 0.001) (5). This finding suggests that HCC patients with PVTT benefit from TACE-S treatment. According to the REFLECT study (7), compared to sorafenib, lenvatinib, exhibits a better overall response rate (ORR) (29.6% vs. 6.9%; *p* < 0.001), progression-free survival (PFS) (7.2 vs. 4.6 months, *p* < 0.001), and non-inferior OS (13.6 vs. 12.3 months). TACE combined with lenvatinib (TACE-L) also presented a significantly longer OS than lenvatinib alone (8). Hence, for HCC with PVTT, TACE-L may have better clinical efficacy than TACE-S. However, there is currently a lack of evidence on TACE-L versus TACE-S. Therefore, we performed this study to compare the efficacy of TACE-L vs. TACE-S in the treatment of HCC with PVTT.

## Methods

**Figure 1:**
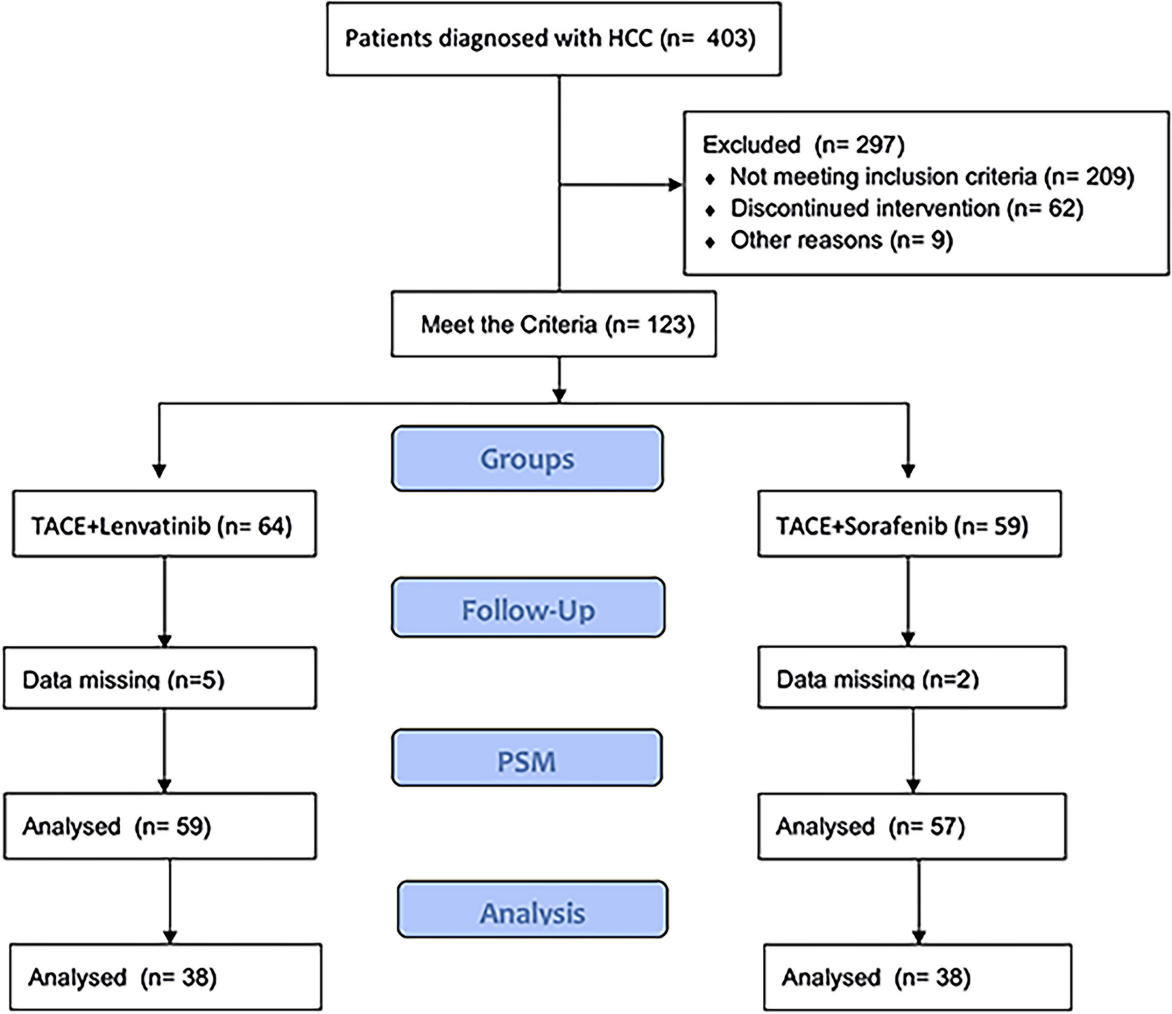
Flow chart for selecting HCC patients with PVTT for this study.

This cohort study was approved by the ethics committee of our institution. The trial registration ID was ChiCTR2100046490 (chictr.org. cn). This study was conducted ethically in accordance with the World Medical Association Declaration of Helsinki. Written informed consent was obtained from every patient. The HCC was diagnosed by histopathological examination. PVTT was diagnosed by magnetic resonance imaging (MRI), abdominal dynamic computer tomography (CT) or ultrasonic examination. All procedures were strictly according to the CONSORT guidelines. Patients were recruited from September 2017 to December 2019. Patients were recruited into two groups according to their willingness. All patients were followed up to February 2021. Among these patients, 59 and 57 were included in the TACE-L and TACE-S groups, respectively (**Figure 1**).

### Eligibility Criteria

All patients were preoperatively evaluated by MRI, abdominal dynamic CT, and/or abdominal ultrasonography. Inclusion criteria were as follows: (1) unresectable HCC with PVTT; (2) international normalized ratio was lower than 1.5; (3) no history of any anti-tumor treatment in the previous 6 months; (4) liver function with Child-Pugh A or B; (5) no serious concurrent medical illness; (6) Eastern Cooperative Oncology Group (ECOG) score ≤ 2; and (7) historically or cytologically proven HCC.

Exclusion criteria: (1) <18 years old or ≥75 years old; (2) esophageal variceal bleeding within the previous 3 months; (3) patients who previously used targeted therapy, systemic chemotherapy, programmed death ligand-1 (PD-L1) inhibitors, PD-L2 inhibitors, cytotoxic T lymphocyte associate protein (CTLA-4) antibody inhibitors, or antitumor Chinese medicines; (4) pregnant females; (5) hepatic artery thrombosis; (6) acute tumor rupture with hemoperitoneum; (7) extrahepatic organs not including regional lymph node metastasis; (8) a history of hepatic encephalopathy; (9) heart failure or concurrent ischemic heart disease; and (10) patients with malignant tumors in other organs.

### Procedures

The treatment protocol was described in a previous study (9). Each patient received the TACE treatment. Briefly, after local anesthesia with 3–5 ml of 1% lidocaine administered in the groin, hepatic arteriography was performed. Then, 2–20 ml of an injectable suspension of iodized oil (10-20 ml, Guerbet) and epirubicin (40-45 mg, Pfizer) was injected into the target artery using a 2-F microcatheter (Terumo), followed by gelatin sponge particle (350–560 μm, Alicon) embolization until arterial flow stasis was achieved. Treatment was continued until progression to the TACE refractory criteria, unacceptable toxicity, or withdrawal of consent.

Sorafenib (Pfizer) or lenvatinib (Merck) was administered within 7 days before or after TACE. Drug dosage was based on the recommended doses in the SHARP (10) and REFLECT (7) studies, respectively: sorafenib: 400 mg orally, twice per day; lenvatinib: 8 mg orally, once per day (body weight < 60 kg) or 12 mg orally, once per day (body weight ≥ 60 kg). Doses were adjusted according to the grade of the National Cancer Institute’s Common Terminology Criteria for Adverse Events (CTCAE version 4.0). Lenvatinib or sorafenib was continually administered until disease progression developed or unacceptable adverse events (AEs) appeared.

### Follow-Up and Assessment

After 4 weeks of TACE, tumors were assessed by dynamic CT or MRI every 3 months, with tumor marker tests performed at the same time (11). All patients were followed up by the same multidisciplinary team after treatment. Complete response (CR), partial response (PR), progressive disease (PD), and stable disease (SD) assessment of liver tumors was performed according to the modified Response Evaluation Criteria in Solid Tumors (mRECIST) (12) within 1 month after the first TACE session (13). The disease control rate (DCR) and overall response rate (ORR) were defined as CR/PR/SD and CR/PR, respectively. PFS was defined as the time from patients’ TACE treatment to disease progression. AEs were evaluated using CTCAE version 4.0. OS was calculated as the time from TACE treatment to death or the last follow-up.

### Statistical Analysis

SPSS 20.0 (IBM, USA) and R-studio (version 4.1.2) were used for statistical analysis. Normally distributed data were expressed as the median and 95% confidence interval (CI), and enumeration data were expressed as *n* (%). Independent-samples *t*-test or *χ* ^2^ test was used to assess two groups of baseline characteristics. OS and PFS were evaluated using the Kaplan–Meier method. The Cox model was used to identify risk factors. A *p*-value < 0.05 was defined as statistically significant.

## Results

### Patient Demographics

A total number of 116 patients were recruited in this study (TACE-L, 59; TACE-S, 57). The mean age was 54.05 ± 11.35 years in the TACE-L group and 55.18 ± 10.94 in the TACE-S group. The baseline characteristics of sex, ECOG score, PVTT classification, AFP level, and pre- and post-propensity score matching (PSM) are presented in **Table 1**.

**Table 1:** Baseline characteristics before and after propensity score matching.

Variables	The entire cohort			The PSM cohort		
	TACE-L (*N* = 59)	TACE-S (*N* = 57)	*p*-value	TACE-L (*N* = 38)	TACE-S (*N* = 38)	*p*-value
Age	54.05 ± 11.35	56.18 ± 12.16	0.619	55.18 ± 10.94	54.39 ± 12.17	0.504
Sex			0.501			1.000
Male	54 (91.5)	50 (87.7)		34 (89.5)	34 (89.5)	
Female	5 (8.5)	7 (12.3))		4 (10.5)	4 (10.5)	
Pathogenesis			0.544			0.422
HBV	54 (91.5)	49 (86.0)		36 (94.7)	34 (89.5)	
HCV	1 (1.7)	3 (5.3		0	3 (7.9)	
Unclear	4 (6.8)	5 (8.8)		2 (5.3)	1 (2.6)	
PVTT			0.454			0.763
Vp2	34 (57.6)	29 (50.9)		20 (52.6)	20 (52.6)	
Vp3	16 (27.1)	17 (29.8)		11 (28.9)	13 (34.2)	
Vp4	9 (15.3)	11 (19.3)		7 (18.4)	5 (13.2)	
Local Lymphatic metastasis			0.109			1.000
Absent	45 (76.3)	50 (87.7)		32 (84.2)	32 (84.2)	
Present	14 (23.7)	7 (12.3)		6 (15.8)	6 (15.8)	
Recurrence			0.684			0.761
Absent	48 (81.4)	48 (84.2)		31 (81.6)	32 (84.2)	
Present	11 (10.2)	9 (15.8)		7 (18.4)	6 (15.8)	
TACE times	2.03 ± 0.118	2.09 ± 0.198	0.284	1.98 ± 0.136	2.18 ± 0.25	0.612
Size			0.742			0.629
<7 cm	19 (32.2)	20 (35.1)		14 (36.8)	12 (31.6)	
>7 cm	40 (67.8)	37 (64.9)		24 (63.2)	26 (68.4)	
Number of Tumor			0.664			0.479
<3	36 (61.0)	37 (64.9)		22 (57.9)	25 (65.8)	
≥3	23 (39.0)	20 (35.1)		16 (42.1)	13 (34.2)	
Tumor Location			0.959			1.000
Right Lobe	34 (57.6)	32 (56.1)		19 (50.0)	20 (52.6)	
Left Lobe	5 (8.5)	4 (7.0)		3 (7.9)	3 (7.9)	
Both Lobe	20 (33.9)	21 (36.8)		16 (42.1)	15 (39.5)	
HBV Amplification			0.122			0.788
Absent	10(16.9)	14 (24.6)		7 (18.4)	8 (21.1)	
Present	34 (57.6)	22 (38.6)		20 (52.6)	17 (44.7)	
Unknown	15 (25.4)	21 (36.8)		11 (28.9)	13 (34.2)	
ECOG			0.804			0.446
0	7 (11.9)	6 (10.5)		7 (18.4)	3 (7.9)	
1	35 (59.3)	34 (59.6)		20 (52.6)	24 (63.2)	
2	15 (25.4)	14 (24.6)		11 (28.9)	9 (23.7)	
3	2 (3.4)	3 (5.3)		0	2 (5.3)	
APT (s)			0.938			1.000
≤40	4 (6.8)	4 (7.4)		3 (7.9)	3 (7.9)	
40–75,000	47 (79.7)	44 (81.5)		29 (76.3)	30 (78.9)	
>75,000	8 (13.6)	6 (11.1)		6 (15.8)	5 (13.2)	
Child-Pugh			0.677			1.000
A	56 (94.9)	55 (96.5)		37 (97.4)	37 (97.4)	
B	3 (5.1)	2 (3.5)		1 (2.6)	1 (2.6)	
Ascites			0.235			1.000
Absent	59 (100.0)	55 (96.4)		38 (100)	38 (100.0)	
Present	0 (0.0)	2 (3.6)		0 (0.0)	0 (0.0)	
AFP (ng/ml)			0.685			0.813
<400	26 (44.1)	23 (40.4)		14 (36.8)	15 (39.5)	
≥400	33 (55.9)	34 (59.6)		24 (63.2)	23 (60.5)	
Hemoglobin (g/L)	138.79 ± 20.58	137.53 ± 23.84	0.301	138.88 ± 2.86	141.61 ± 19.73	0.845
WBC	5.96 ± 0.417	5.67 ± 0.33	0.766	5.76 ± 0.33	6.16 ± 3.650	0.731
PLT	172.59 ± 13.12	159.96 ± 11.04	0.662	182.85 ± 17.03	181.76 ± 99.26	0.955
ALT	50.10 ± 4.05	42.94 ± 4.31	0.647	54.50 ± 5.43	47.13 ± 27.85	0.572
AST	71.66 ± 5.95	61.02 ± 4.52	0.100	74.20 ± 8.06	65.26 ± 36.24	0.422

HBV, Hepatitis B virus; HCV, Hepatitis C virus; PVTT, Portal vein tumor thrombus; ECOG, Eastern Cooperative Oncology Group; AFP, Alpha fetoprotein; WBC, White blood cells; PLT, Platelet Count; ALT, Alanine transaminase; AST, Aspartate aminotransferase.

### Overall Survival

**Figure 2:**
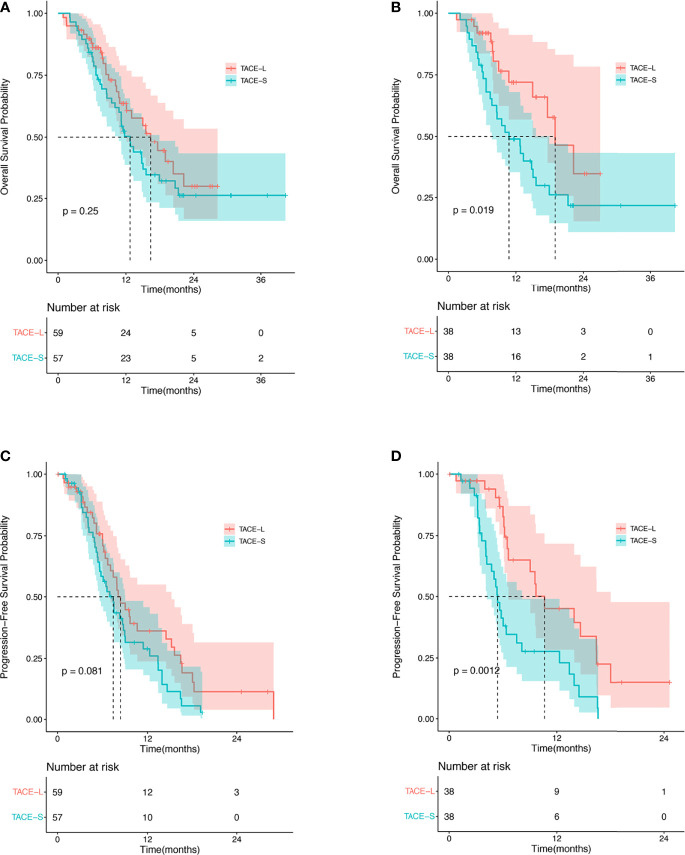
Kaplan–Meier analysis for OS before PSM **(A)**, OS after PSM **(B)**, PFS before PSM **(C)**, and PFS after PSM **(D)**.

**Figure 3:**
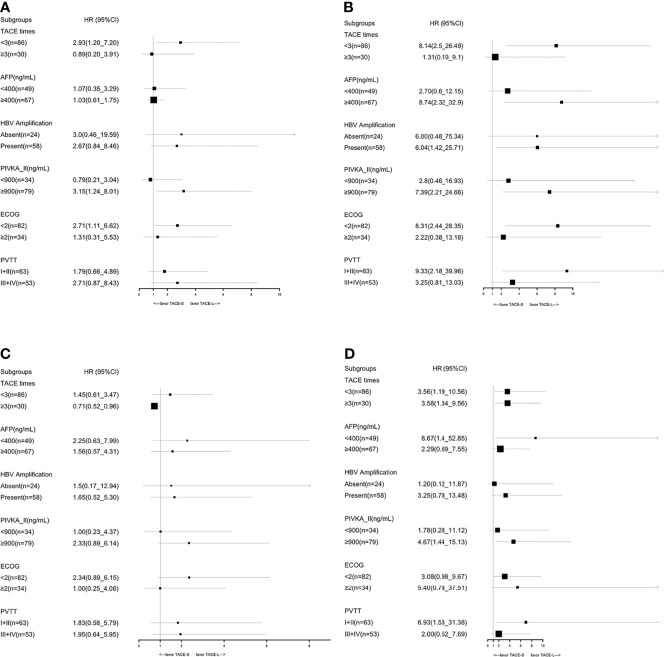
Subgroup analyses of OS before PSM **(A)**, OS after PSM **(B)**, PFS before PSM **(C)**, and PFS after PSM **(D)** for known prognostic factors.

In the TACE-L and TACE-S groups, the median OS was 16.4 months (95% CI: 10.93–21.81 months) and 12.7 months (95% CI: 10.8–17.9 months), respectively (HR 1.34; 95% CI: 0.81–2.20; *p* = 0.25) (**Figure 2**). At 6, 12, and 24 months, the estimated OS rates were 81.4% vs. 78.9%, 40.7% vs. 40.4%, and 8.5% vs. 8.8% in the TACE-L and TACE-S groups, respectively (**Supplementary Table 1**). In the subgroup analysis, based on the number of TACE procedures (<3 times), the median OS was 14.97 months (95% CI: 9.49–20.45 months) vs. 11.5 months (95% CI: 10.09–12.92 months) (HR 2.93; 95% CI: 1.27–2.00; *p* = 0.017). For patients with α-fetal protein (AFP) ≥ 400 ng/ml, the median OS was 17.6 months (95% CI: 9.64–25.56 months) vs. 11.5 months (95% CI: 9.35–13.65 months) (HR 1.03; 95% CI: 0.61-1.75; *p* = 0.009). The median OS was 20.37 months (95% CI: 13.78–26.96 months) vs. 14.8 months (95% CI: 11.76–17.84 months) (*p* = 0.433) in the subgroup of ECOG < 2 (HR 2.71; 95% CI: 1.11–6.62; *p* = 0.027) (**Figure 3**).

After PSM, the median OS was 18.97 months (95% CI: 13.53–24.41 months) and 10.77 months (95% CI: 6.07–15.47 months) in the TACE-L and TACE-S groups, respectively (HR 2.21; 95% CI: 1.12–4.38; *p* = 0.022) (**Figure 2**). The estimated OS rates at 6, 12, 24, and 36 months were 81.6% vs. 73.7%, 34.2% vs. 42.1%, and 8.9% vs. 5.3% in the TACE-L and TACE-S groups, respectively. In the subgroup analysis, the median OS of Vp2 PVTT was 22.23 months (95% CI: 12.78–31.61 months) vs. 12.8 months (95% CI: 8.01–17.59 months) (HR 9.33; 95% CI: 2.18–28.35; *p* < 0.001) in TACE-L compared to TACE-S, respectively. Based on the number of TACE procedures (<3 times), the median OS was 14.97 months vs. 10.77 months (HR 8.14; 95% CI: 2.53-26.49; *p* < 0.001). For patients with AFP ≥ 400 ng/ml, the median OS was 22.23 months vs. 10.77 months (HR 8.74; 95% CI: 2.32–32.90; *p* = 0.001). The median OS was 22.23 months vs. 12.8 months in subgroup of ECOG < 2 (HR 8.31; 95% CI: 2.44–28.35; *p* < 0.001) (**Figure 3**).

### Progression-Free Survival

The median PFS time was 8.4 months (95% CI: 7.07–15.2 months) and 7.43 months (95% CI: 5.63–9.03 months) in the TACE-L and TACE-S groups, respectively (HR 1.54; 95% CI: 0.98–2.41; *p* = 0.081) (**Figure 2**). The estimated PFS rates at 6, 12, and 24 months were 59.3% vs. 42.1%, 20.3% vs. 17.5%, and 3.4% vs. 1.8% in the TACE-L and TACE-S groups, respectively (**Supplementary Table 1**). Before PSM, the subgroup analysis showed no difference in PVTT, the number of TACE procedures or ECOG.

After PSM, the median PFS time was 10.6 months (95% CI: 6.6–18.0 months) and 5.4 months (95% CI: 4.2–8.1 months) in the TACE-L and TACE-S groups, respectively (HR 2.62; 95% CI: 1.43–4.80; *p* = 0.002) (**Figure 2**). The estimated PFS rates at 6, 12, and 24 months were 52.6% vs. 34.2%, 23.7% vs. 15.8%, and 2.6% vs. 0% in the TACE-L and TACE-S groups, respectively. In the subgroup analysis based on PVTT classification, the median PFS of Vp2 PVTT was 9.67 months (95% CI: 8.76–10.58 months) vs. 5.33 months (95% CI: 4.38–6.28 months) (HR 6.93; 95% CI: 1.53-31.34; *p* < 0.001) in TACE-L vs. TACE-S, respectively. Based on the number of TACE procedures < 3 times, the median PFS was 9.03 months (95% CI: 4.68–13.38 months) vs. 5.2 months (95% CI: 3.77–6.63 months) (HR 3.56; 95% CI: 1.19–10.59; *p* = 0.02). For the number of TACE procedures ≥ 3 times, the median PFS was 16.4 months (95% CI: 7.80–25.00 months) vs. 6.37 months (95% CI: 5.50–7.24 months) (HR 1.99; 95% CI: 0.66–5.98; *p* = 0.222). The median PFS was 10.63 months (95% CI: 6.06–15.20 months) vs. 9.69 months (95% CI: 3.11–5.29 months) in the subgroup of patients with AFP < 400 ng/ml (HR 8.67; 95% CI: 1.40–53.85; *p* = 0.021) (**Figure 3**).

### Tumor Response

A total of 56 and 54 patients were evaluated for tumor response in the TACE-L and TACE-S groups. Comparing TACE-L and TACE-S, the CR was 10.71% vs. 5.56% (OR 2.04; 95% CI: 0.48–8.61; *p* = 0.49); the PR was 50.5% vs. 33.3% (OR 2.00; 95% CI: 0.96–4.32; *p* = 0.076); ORR was 60.7% vs. 38.9% (OR 2.43; 95% CI: 1.13–5.23; *p* = 0.022); the DCR was 96.4% vs. 96.3% (OR 1.04; 95% CI: 0.14–7.65; *p* = 1.00) (**Table 2**).

**Table 2:** The imaging response assessed using mRECIST criteria.

Variable	Group	The entire cohort				The PSM cohort		
		Percent		Odds Ratio	*p*-value	Percent	Odds Ratio	*p*-value
CR				2.04 (0.48–8.61)	0.490		1.88 (0.32–10.99)	0.677
	TACE-L	10.71 (6/56)				10.53 (4/38)		
	TACE-S	5.56 (3/54)				5.56 (2/36)		
PR				2.00 (0.96–4.32)	0.076		2.89 (1.10–7.61)	0.030
	TACE-L	50.0 (28/56)				56.23 (20/38)		
	TACE-S	33.3 (18/54)				27.78 (10/36)		
SD				0.41 (0.19–0.89)	0.023		0.29 (0.11–0.76)	0.011
	TACE-L	35.7 (20/56)				34.21 (13/38)		
	TACE-S	57.4 (31/54)				63.89 (23/36)		
PD				0.96 (0.13–7.09)	1.000		0.95 (0.06–15.71)	1.000
	TACE-L	3.57 (2/56)				2.63 (1/38)		
	TACE-S	3.7 (2/54)				2.78 (1/36)		
ORR				2.43 (1.13–5.23)	0.022		2.69 (1.05–6.90)	0.037
	TACE-L		60.7 (34/56)			66.8 (24/38)		
	TACE-S	38.9 (21/54)				33.3 (12/36)		
DCR				1.04 (0.14–7.65)	1.000		1.06 (0.06–17.56)	1.000
	TACE-L	96.4 (54/56)				97.4 (37/38)		
	TACE-S	96.3 (52/54)				97.2 (35/36)		
Overall					0.030			0.017

CR, Complete response; PR, Partial response; SD, Stable disease; PD, Progressive disease; ORR, Overall response rate; DCR, Disease control rate.

After PSM, the CR was 10.53% vs. 5.56% (OR 0.85; 0.32–10.99; *p* = 0.67), and the PR was 56.23% vs. 27.78% (OR 2.89; 1.10–7.61; *p* = 0.03) (**Table 2**). The ORR was 66.8% vs. 33.3% (OR 0.85; 1.05–6.90; *p* = 0.037) (**Table 2**), and the DCR was 97.4% vs. 97.2% (OR 1.06; 95% CI: 0.06–17.56; *p* = 1.00).

### Univariable and Multivariable Analyses of OS in the PSM Cohort

Additional univariable and multivariable Cox regression analyses with robust estimators were performed in the PSM cohort (**Table 3**). TACE frequency < 3 (HR, 0.39; 95% CI: 0.17–0.9, *p* = 0.028), ECOG < 2 (HR, 1.96; 95% CI: 1.02–1.75, *p* = 0.045), and treatment method (HR, 2.21; 95% CI: 1.12–4.38, *p* = 0.02) were critically important factors for longer overall survival.

**Table 3:** Univariate and multivariate analysis of overall survival.

Risk factor	The entire cohort				The PSM cohort			
	Univariate Analysis		Multivariate Analysis		Univariate Analysis		Multivariate Analysis	
	HR (95% CI)	*p*-value	HR (95% CI)	*p*-value	HR (95% CI)	*p-*value	HR (95% CI)	*p*-value
Age level (<60/≥60)	0.7 (0.41–1.21)	0.206			0.99 (0.51–1.95)	0.987		
Gender (M/F)	0.99 (0.42–2.29)	0.971			1.16 (0.41–3.26)	0.786		
Pathogenesis								
HBV	Reference	0.127	2.14 (0.97–4.71)	0.06	Reference	0.723		
HCV	2.03 (0.63–6.55)	0.236	1.98 (0.87–4.52)	0.106	1.41 (0.33–5.98)	0.642		
Unclear	0.39 (0.12–1.25)	0.114			0.52 (0.07–3.82)	0.523		
Type of PVTT (I+II/III+IV)	1.12 (0.68–1.82)	0.658			1.12 (0.6–2.12)	0.718		
Local lymphatic metastasis (no/yes)	1.04 (0.55–1.95)	0.907			1.1 (0.46–2.62)	0.835		
Recurrence (no/yes)	0.89 (0.47–1.67)	0.708			1.09 (0.5–2.39)	0.825		
TACE frequency (<3, ≥3)	0.34 (0.18–0.65)	0.001	0.34 (0.18–0.66)	0.001	0.39 (0.17–0.9)	0.028	0.35 (0.15–0.82)	0.016
Size (<7/≥7)	1.56 (0.91–2.7)	0.109			1.28 (0.66–2.49)	0.474		
Number of Tumor	1.38 (0.84–2.26)	0.202			1.56 (0.83–2.94)	0.165		
Tumor Location								
Right Lobe	Reference	0.265			Reference	0.592		
Left Lobe	0.72 (0.22–2.36)	0.592			0.85 (0.2–3.65)	0.824		
Both Lobe	1.44 (0.87–2.4)	0.158			1.36 (0.71–2.59)	0.354		
HBV DNA								
Absent	Reference	0.043			Reference	0.287		
Present	2.39 (1.1–5.19)	0.028			1.88 (0.7–5.02)	0.208		
Unknown	2.76 (1.23–6.17)	0.014			2.28 (0.82–6.33)	0.114		
ECOG (<2/≥2)	2.31 (1.37–3.9)	0.002	2.06 (1.21–3.5)	0.008	1.96 (1.27–3.8)	0.045	1.63 (0.8–3.33)	0.178
APT (s)								
≤40	Reference	0.482			Reference	0.644		
40 < x ≤ 75,000	0.63 (0.25–1.59)	0.329			0.61 (0.22–1.75)	0.361		
>75,000	0.88 (0.28–2.77)	0.826			0.72 (0.19–2.69)	0.628		
Child-Pugh (A/B)	2.37 (0.94–5.99)	0.067			2.89 (0.68–12.27)	0.149		
AFP (<400/≥400 ng/ml)	1.36 (0.82–2.27)	0.236			0.98 (0.51–1.9)	0.96		
PIVKA-II (<900/≥900, ng/ml)	1.58 (0.9–2.78)	0.108			2.06 (0.97–4.39)	0.061	2.26 (0.99–5.15)	0.053
Hemoglobin (<100/≥100)	0.26 (0.09–0.74)	0.001			0.39 (0.09–1.66)	0.203		
WBC (<4/≥4^12^/L)	0.67 (0.39–1.17)	0.161			0.69 (0.34–1.39)	0.297		
PLT (<100/≥100^9^/L)	1.11 (0.61–2.03)	0.730			1.52 (0.67–3.46)	0.321		
ALT (<35/≥35 IU/L)	1.02 (0.61–1.7)	0.944			1.3 (0.68–2.48)	0.426		
AST (<35/≥35 IU/L)	1.02 (0.5–2.08)	0.953			0.99 (0.41–2.37)	0.976		
Treatment (TACE-L/TACE-S)	0.31 (0.79–2.13)	1.293			2.21 (1.12–4.38)	0.022	1.93 (0.96–3.88)	0.660

M, Male; F, Female; HBV, Hepatitis B virus; HCV, Hepatitis C virus; PVTT, Portal vein tumor thrombus; ECOG, Eastern cooperative oncology group; PIVKA-II, Protein Induced by Vitamin K absence or antagonist-II; AFP, Alpha fetoprotein; WBC, White blood cells; PLT, Platelet count; ALT, Alanine transaminase; AST, Aspartate aminotransferase.

### Adverse Events

No treatment-related death was observed in either group (**Table 4**). One patient treated with TACE-S experienced cholecystitis and recovered through surgical resection. One patient treated with TACE-L had a liver abscess after the first chemoembolization, whose condition was improved after puncture drainage and antibiotics treatment. In the TACE-L group, a total of 57 complications were observed in 38 patients. The most frequent adverse events were hand–foot syndrome and diarrhea (17.5%). Grade 3–4 toxicities were observed in 13 (24.0%) patients. Three patients (7.9%) stopped using lenvatinib due to intolerance. Fifty-two complications were observed in 40 patients in the TACE-S group. The highest incidences were hand–foot syndrome (30.7%) and diarrhea (25.0%). Grade 3–4 toxicities were observed in 10 patients (17.50%). Two patients (5.0%) stopped using sorafenib due to intolerance.

**Table 4:** Adverse event categories before propensity score matching.

	TACE-L (*N* = 59)			TACE-S (*N* = 57)		
	Total (*N*%)	Grade 3	Grade 4	Total (*N*%)	Grade 3	Grade 4
Thrombocytopenia	0	0	0	1 (1.9)	0	1
Transaminitis	5 (8.7)	1	0	3 (5.8)	0	0
Hyperbilirubinemia	2 (3.5)	1	0	1 (1.9)	0	0
Hypertension	8 (14.0)	2	0	4 (7.7)	2	0
Oral ulcer	4 (7.0)	1	0	1 (1.9)	0	0
Bleeding	5 (8.7)	1	0	3 (5.8)	0	0
Diarrhea	10 (17.5)	2	0	13 (25.0)	2	0
Rash	3 (5.2)	0	0	4 (7.7)	0	0
Hand foot skin reaction	10 (17.5)	2	0	16 (30.7)	3	0
Nausea	8 (14.0)	2	0	4 (7.7)	0	0
Trachyphonia	1 (1.7)	1	0	1 (1.9)	0	0
Liver abscess	1 (1.7)	0	0	0	0	0
Cholecystitis	0	0	0	1 (1.9)	1	0
Total	57	13	0	52	9	1

## Discussion

In the treatment of HCC with PVTT, accumulating evidence suggests that TACE-S has a higher OS rate than mono-sorafenib. Recently, the REFLECT study (7) showed that lenvatinib was superior to sorafenib with respect to PFS. Moreover, lenvatinib has a 64.87% cost-saving advantage than sorafenib (14). Ding et al. have reported TACE-L vs. TACE-S in HCC patients (15). Our study offers a comparison of the efficacy between TACE-L and TACE-S in HCC patients with PVTT.

Previously, several studies have demonstrated that lenvatinib is superior to sorafenib in PFS (*p* = 0.009) and TTP (*p* = 0.005) with a similar result in OS (HR 0.92, 95% CI: 0.79–1.06) (7). Our results revealed that TACE-L achieved significantly better benefits with respect to PFS, OS, and ORR than TACE-S in HCC patients with PVTT. These results are similar to those of a previously published study (15), in which TACE-L was better than TACE-S in HCC with PVTT in terms of median TTP (4.7 vs. 3.1 months, *p* = 0.037) and numerically better median OS (15.6 vs. 10.8 months, *p* = 0.15) and ORR (50.0 vs. 25.0%, *p* = 0.07). In our study, we did not observe any significant differences in either OS or PFS in the entire cohort but only after PSM. Subgroup analysis showed that TACE-L achieved a better PFS and OS than TACE-S in Vp2 PVTT and procedure times < 3.

For sorafenib treatment, TACE-S exhibited a better PFS benefit than TACE alone (*p* < 0.01) (16). Furthermore, Peng et al. (5) demonstrated that patients with PVTT had a better median OS (*p* < 0.001) and TTP (*p* < 0.001) in the TACE-S group than in the monosorafenib group. This is similar to a previous study reporting that TACE-S is superior to sorafenib with respect to TTP and OS (17). Furthermore, another study reported a significantly improved TTP (*p* = 0.003), PFS (*p* = 0.01), and ORR (*p* = 0.005) (18). Wu et al. (19) reported no difference between TACE-S and sorafenib in OS (*p* = 0.223). A similar study reported that TACE-S cannot improve OS compared to sorafenib (HR 0.91; 90% CI 0.69–1.21; *p* = 0.29) in patients with advanced HCC (20).

Only a few studies have tested the combination of TACE with lenvatinib. Previously, Zhang et al. (21) presented a DCR (92.3%) in TACE-L treatment of HCC with PVTT. This is similar to the DCR (97.4%) observed in our study. Chen et al. (22) demonstrated that the median disease-free survival was 12.0 months in the TACE-L group, which is similar to another study (8). He et al. (23) compared lenvatinib alone to transcatheter arterial infusion plus lenvatinib or toripalimab. The combination group achieved better PFS (11.1 versus 5.1 months, *p* < 0.001), OS (*p* < 0.001), and ORR (59.2% versus 9.3%, *p* < 0.001). The combination group reported a similar PFS and ORR to the TACE-L group in our study.

Compared to TACE-S, TACE-L resulted in a higher incidence of grade ≥3 adverse events in our study. This is entirely contradictory to a previous study (7), which reported fewer grade ≥3 adverse events with lenvatinib treatment. Similar to a previous study (24), no differences in serious adverse events or treatment-related deaths were observed between the TACE-L and TACE-S groups in our study. In another study, the most frequent adverse events were hypertension (17%) and proteinuria (12.2%) (8). However, the most frequent adverse events in our study were hand–foot syndrome and diarrhea. These differences might arise from different procedures. The previous study used both TACE with epirubicin and/or cisplatin and performed hepatic arterial infusion chemotherapy with cisplatin and 5-Fu (8).

## Limitations

There are several limitations in this study that must be addressed. Although we performed PSM, this method does not entirely eliminate selection bias. This study had a relatively small sample size and did not allow us to do some analyses of subgroups. Especially after PSM, the sample size was further decreased. Hence, a large-scale and randomized study is needed.

## Conclusions

Both TACE-L and TACE-S are safe, well-tolerated treatments for HCC patients with PVTT. For HCC patients with PVTT, TACE-L may be significantly superior to TACE-S with respect to OS, PFS, and ORR. A larger-scale randomized clinical trial is needed.

## Data Availability Statement

The datasets presented in this study can be found in online repositories. The names of the repository/repositories and accession number(s) can be found at: www.medresman.org.

## Ethics Statement

The studies involving human participants were reviewed and approved by West China Hospital. The patients/participants provided their written informed consent to participate in this study.

## Author Contributions

All authors listed have made a substantial, direct, and intellectual contribution to the work and approved it for publication.

## Funding

This study has received funding from the Department of Science and Technology of Sichuan Province of China (21YYJC2782), Health Commission of Sichuan Province (20PJ043), and Post-Doctor Research Project, West China Hospital, Sichuan University (2021HXBH023).

## Conflict of Interest

The authors declare that the research was conducted in the absence of any commercial or financial relationships that could be construed as a potential conflict of interest.

## Publisher’s Note

All claims expressed in this article are solely those of the authors and do not necessarily represent those of their affiliated organizations, or those of the publisher, the editors and the reviewers. Any product that may be evaluated in this article, or claim that may be made by its manufacturer, is not guaranteed or endorsed by the publisher.
