# Supplementary material for: TACE Plus Lenvatinib Versus TACE Plus Sorafenib for Unresectable Hepatocellular Carcinoma With Portal Vein Tumor Thrombus: A Prospective Cohort Study
Source: Front Oncol. 2021 Dec 23;11:821599. doi: 10.3389/fonc.2021.821599 (PMC8733478; doi:10.3389/fonc.2021.821599)
Supplement: Supplementary file 1 [file Table_1.doc]

**Table S1** The progression-free survival and overall survival analysis

| N(%) |  | The entire cohort | | | | The PSM cohort | | | | |  |  |
| --- | --- | --- | --- | --- | --- | --- | --- | --- | --- | --- | --- | --- |
|  | TACE-L (N=59) | | TACE-S (N=57) | OR 95%CI | p | | TACE-L (N=38) | TACE-S (N=38) | OR 95%CI | p | | |
| Median OS, 95% CI, months | | |  |  |  | |  |  |  |  | | |
| 6-months OS rate | 48 (81.4%) | | 45 (78.9%) | 1.16 (0.47-2.90) | 0.745 | | 31 (81.6%) | 28 (73.7%) | 1.58 (0.53-4.72) | 0.409 | | |
| 1-year OS rate | 24 (40.7%) | | 23 (40.4%) | 1.01 (0.48-2.13) | 0.971 | | 13 (34.2%) | 16 (42.1%) | 0.72 (0.28-1.81) | 0.479 | | |
| 2-year OS rate | 5 (8.5%) | | 5 (8.8%) | 0.96 (0.26-3.52) | 1.000 | | 3 (7.9%) | 2 (5.3%) | 1.54 (0.24-9.80) | 1.000 | | |
| Median PFS, 95% CI, months | | |  |  |  | |  |  |  |  | | |
| 6-months PFS rate | 35 (59.3%) | | 24 (42.1%) | 2.01(0.96-4.20) | 0.064 | | 20 (52.6%) | 13 (34.2%) | 2.14 (0.85-5.39) | 0.105 | | |
| 1-year PFS rate | 12 (20.3%) | | 10 (17.5%) | 1.20 (0.47-3.05) | 0.701 | | 9 (23.7%) | 6 (15.8) | 1.66 (0.53-5.22) | 0.387 | | |
| 2-year PFS rate | 2 (3.4%) | | 1 (1.8%) | 1.97(0.17-22.29) | 1.000 | | 1 (2.6%) | 0 (0%) | - | - | | |

Abbreviation: OS, Overall survival; CI, Cconfidence interval
